# Challenges for the determination of spiramycin in aqueous matrices using LC-MS/MS: evidence for the solvent intrusion on the molecule integrity

**DOI:** 10.1039/d2ra00205a

**Published:** 2022-06-09

**Authors:** Azadeh Nasiri, Shaya Mokhtari, Reza Jahani, Bahram Daraie, Hassan Yazdanpanah, Mehrdad Faizi, Farzad Kobarfard

**Affiliations:** Department of Pharmacology and Toxicology, School of Pharmacy, Shahid Beheshti University of Medical Sciences Tehran Iran; Food Safety Research Center, Shahid Beheshti University of Medical Sciences Tehran Iran; Central Research Laboratories, Shahid Beheshti University of Medical Sciences Tehran Iran farzadkf@yahoo.com kobarfard@sbmu.ac.ir; Phytochemistry Research Center, Shahid Beheshti University of Medical Sciences Tehran Iran; Department of Medicinal Chemistry, School of Pharmacy, Shahid Beheshti University of Medical Sciences Tehran Iran

## Abstract

Liquid chromatography-tandem mass spectroscopy (LC-MS/MS) is an accurate and specific technique for drug residue analysis in different matrices. The high specificity and sensitivity of the multiple reaction monitoring (MRM) approach for detecting drugs such as aldehydes, which have the potential to change mass during the sample preparation phase, becomes a drawback during the analysis process. In this study, concerns about the intrusion of solvent molecules into spiramycin's chemical structure as an aldehydic drug as well as the stability of spiramycin in the milk matrix were addressed. Furthermore, the binding sites where the solvent molecules could bind to spiramycin molecules were investigated through nuclear magnetic resonance (NMR) spectroscopy. It was revealed that water, ethanol, and methanol as protic solvents can add to the formyl group of spiramycin molecules during standard solutions preparation while there was no evidence for the addition of acetonitrile and dimethyl sulfoxide (aprotic solvents). In addition, as time passed, the peak area of spiramycin decreased either in the spiked aqueous sample or milk sample while an increase in the peak area of H_2_O-bound spiramycin was observed. After 96 h, more than 90% of spiramycin was converted to H_2_O-bound spiramycin. In conclusion, we can propose the use of aprotic solvents for the preparation of spiramycin standard solutions especially when the prepared solutions are not used instantly. Moreover, ion transitions for both spiramycin and its H_2_O-added form (843.6 *m*/*z* to 173.9 *m*/*z* and 861.5 *m*/*z* to 173.9 *m*/*z*, respectively) should be considered for the accurate quantification of spiramycin residue in aqueous samples such as milk.

## Abstract 2

Water, ethanol, and methanol as protic solvents can add to the formyl group of spiramycin molecules during standard solutions preparation while there was no evidence for the addition of acetonitrile and dimethyl sulfoxide as aprotic solvents.

## Graphical abstract

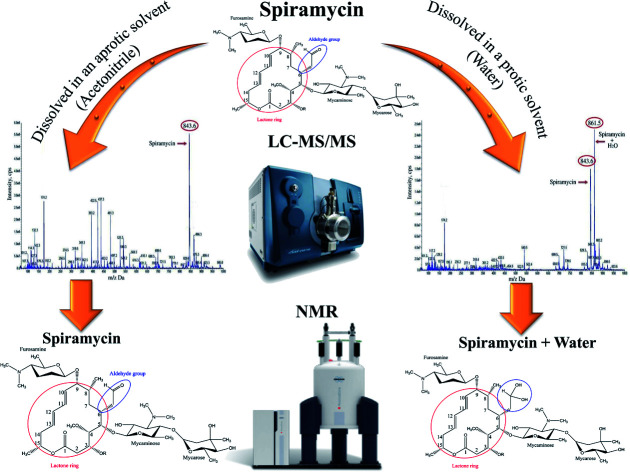

## 1. Introduction

High-performance liquid chromatography-tandem mass spectrometry (HPLC-MS/MS) has become a very common technique to analyze drug residues in different matrices, especially for multi-residue analysis purposes.^1^ The integration of mass spectrometers as the detector to the chromatographic systems offers several advantages such as improved selectivity and specificity which enables the determination of analytes in complex mixtures. Liquid chromatography-tandem mass spectrometry (LC-MS/MS) allows for the measurement of multiple analyte molecules in a single run and multiple reaction monitoring (MRM) is the monitoring method of choice for this purpose. The technique uses a tandem mass strategy that firstly targets the ion corresponding to the intact molecular weight of the compound of interest with subsequent fragmentation of that target ion to produce a group of daughter ions. One of these fragment daughter ions can be selected for quantification purposes. Only compounds with specific parent ions and specific daughter ions corresponding to the mass of the molecule of interest are isolated by the mass spectrometer. By ignoring all other ions that flow into the mass spectrometer, the technique gains high sensitivity and selectivity. In the case of complex samples, it is common to observe co-elution of the mixture's components, resulting in an overlap of signal peaks observed in the total mass spectra. In such cases, the MRM approach is often the most reliable means of solving the problem. The high specificity which is achieved by monitoring the specific ion transitions and the capability of tandem mass spectrometry in simultaneous monitoring of multiple ion transitions enables this technique to generate signals for the compounds even if the chromatographic system fails to separate the analytes and they co-elute from the chromatograph. The multiple reaction monitoring approaches can well eliminate intruders and make the analysis easier.^2^

Although the LC-MS/MS method in the MRM mode increases the sensitivity and specificity of the analysis, it has some pitfalls. For instance, if the chemical structure of the analyte changes in any steps of sample preparation, extraction, dissolution, *etc.*, the *m*/*z* of the analyte will subsequently change and the MRM mode will lose its efficiency to monitor the target analyte. Normally, an MS/MS analysis method is optimized based on the chemical structure of the molecule, and the corresponding precursor-to-product ion transitions and optimal ionization conditions are obtained during the optimization of the analyte signal. In the MRM mode, the detection is based on specific ion transitions, and if the structure of the target analyte changes, the ion transitions will subsequently change. Since the new transition is not targeted, neither the original molecule nor the new derivative will be observed.^3^ Such a state will lead to false-negative results, meaning that the molecule exists but is not detected. In the case of false negatives, if we are looking for a banned substance that presents in the matrix but is not detected, the sample is reported acceptable while that compound or one of its derivatives is present in the matrix, and there is no guarantee that the derivative is less dangerous than the original compound.^4^ The use of stable isotope-labeled analogs of the targeted analytes with identical extraction properties is a complementary strategy to solve this problem.^5^ However, in some multi-residues analyses, only one isotope-labeled internal standard is used for the quantification of different analytes.^6,7^ It should be noted that, if an ultraviolet or fluorescent detector is used, although the previous signal may disappear or lose intensity, a new signal will appear elsewhere in the chromatogram, indicating that the chemical structure of the target analyte, at least in a part, has been converted into something else. In this case, the advantage of MRM specification turns into a disadvantage. This may be the case for some reactive compounds containing aldehyde groups in their structure such as spiramycin. There would be a possibility for change in the molecular mass of spiramycin following intrusion of most commonly used solvents including water, ethanol, methanol, dimethyl sulfoxide (DMSO), and acetonitrile into spiramycin chemical structure. Besides, water is the most common matrix in agricultural and livestock products for the study of contaminants.

**Fig. 1:**
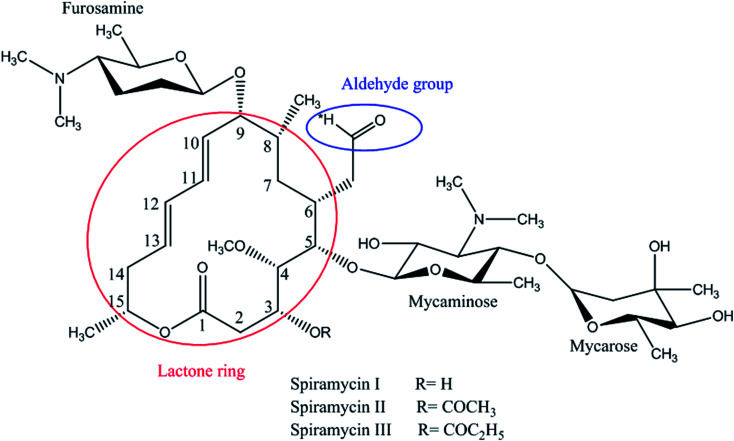
Spiramycin structure and its functional groups.

Spiramycin, an important veterinary drug in China, Europe, and some other countries, is a macrolide antimicrobial agent isolated from *Streptomyces ambofaciens*.^8^ Spiramycin is used for the treatment and control of some bacterial infections in animals.^9^ Spiramycin is a complex of a 16-membered lactone ring and 3 sugars: 2 aminosugars; mycaminose and furosamine and a neutral sugar, mycarose. This macrolide antibiotic is a mixture of 3 compounds, spiramycin I (major component), spiramycin II and spiramycin III (Fig. 1).^10,11^

Spiramycin residue in animal products such as milk can cause serious undesirable effects on consumer health. The Codex Alimentarius Commission has set a maximum residue limit (MRL) for spiramycin in cow's milk to ensure human food safety. Therefore, accurate quantification of spiramycin at the established MRL is an important issue. There are some published LC-MS/MS methods for the determination of spiramycin in different matrices.^12–15^ However, they have not considered the possibility of solvent intrusion into spiramycin chemical structure which may be a source of error in the quantification of spiramycin. The current study was carried out based on the structural features of spiramycin. The present study investigated the possibility of the change in the molecular mass of spiramycin due to the intrusion of most commonly used solvents into spiramycin chemical structure during LC-MS/MS analysis. Besides, the location of the solvent intrusion into spiramycin molecules was investigated through nuclear magnetic resonance (NMR) spectroscopy. Finally, the stability of spiramycin in the milk sample as an aqueous matrix at different time intervals was evaluated. The obtained results help to find the most suitable technique and solvent in terms of stock solutions and working standards preparation during the method validation in the LC-MS/MS measurements.

## 2. Materials and methods

### 2.1. Chemicals, reagents, and solutions

Spiramycin analytical standard from *Streptomyces* sp. (molecular weight: 843.05 g mol^−1^, purity ≥ 90%), deuterated dimethyl sulfoxide (DMSO-d_6_, 99.99%), and spectroscopic grade deuterium oxide (D_2_O) were purchased from Sigma-Aldrich (St. Louis, MO, USA). HPLC grade water, ethanol, methanol, DMSO, and acetonitrile were provided by Merck (Darmstadt, Germany) and used for dissolution of the spiramycin reference standard. Acetic acid (Merck, Darmstadt, Germany), sodium chloride (Merck, Darmstadt, Germany), sodium sulfate (Chem-Lab, Belgium), and sorbent C18 (Chromabond®, GmbH, Germany) were used for sample preparation. Doubly-deionized water was prepared by a Milli-Q water purification system (Millipore, Bedford, MA, USA).

### 2.2. Instrumentation

#### 2.2.1. Liquid chromatography-tandem mass spectrometry

The LC-MS/MS measurement as a highly selective and specific detection technique was carried out to evaluate the possibility of the change in the molecular mass of spiramycin due to solvent intrusion into its chemical structure. To prevent day-to-day variation of the instrumentation, all experiments were carried out within one day when it was possible. Sample analyses were performed on an Agilent 1260 HPLC system (Agilent Technologies, USA) coupled with an AB Sciex 4500 QTRAP mass spectrometer (AB Sciex, USA). The HPLC system was equipped with a Kinetex XB-C18 column (2.6 μm, 100 × 3.0 mm id) (Phenomenex Inc., Torrance, CA, USA). Water containing 0.1% formic acid (solvent A) and acetonitrile containing 0.1% formic acid (solvent B) were used as mobile phases. The LC was operated under an isocratic condition with a composition of 20% mobile phase A and 80% mobile phase B. Column temperature, flow rate, injection volume, and run time were adjusted at 40 °C, 0.6 mL min^−1^, 20 μL, and 5 minutes, respectively.

The mass spectrometer was operating with an electrospray ionization source (ESI) in the positive mode and a triple quadrupole mass analyzer. ESI settings were as follows: turbo ion spray voltage, +5.5 kV; turbo gas temperature, 500 °C; curtain gas pressure, 20 psi; ion source gas 1 pressure: 55 psi; ion source gas 2 pressure: 45 psi; collision gas: medium. Nitrogen was used as the curtain gas and collision gas. Fragmentation was carried out using collision induced dissociation with the settings presented in Table 1. In the ion transition optimization process, full scan mode, product ion scan, and MRM mode were used, respectively. Data acquisition, integration, and quantification were performed using Analyst 1.6.1 software (AB Sciex, USA).

**Table 1:** Ion transitions, collision energy (CE), declustering potential (DP), and entrance potential (EP) used for the detection of spiramycin and H_2_O-bound spiramycin molecules

	Precursor ion (*m*/*z*)	Product ion (*m*/*z*)	CE (V)	DP (V)	EP (V)
Spiramycin	843.6	100.8	128.94	123.16	12
	843.6	173.9^a^	85.89	103.04	15
H_2_O-bound spiramycin	861.5	100.8	37.16	123.16	14
	861.5	173.9^a^	43.86	70.07	14

a Refers to the selected quantifier ions.

##### 2.2.1.1 Evaluation of solvent intrusion into spiramycin

A standard aqueous solution of spiramycin (5 mg L^−1^) was prepared and introduced to the mass system (positive mode) at different time intervals (0 min, 30 min, and 10 h following preparation of the standard solution). The mass spectra were acquired in the full-scan mode. To evaluate the possibility of solvent addition, spiramycin pure standard was dissolved in two other protic solvents *i.e.* methanol and ethanol, and two aprotic solvents *i.e.* acetonitrile and DMSO at the concentration level of 5 mg L^−1^. The standard solutions were directly injected into the mass system, 10 h after the preparation of each reference standard.

##### 2.2.1.2 Stability of spiramycin in the milk matrix

A total of 20 mL of milk sample was spiked with spiramycin at the level of 5 mg L^−1^ and the procedure of sample preparation was carried out at different time intervals (0, 15, 24, 48, 72, and 96 h following spiking of the milk sample). The targeted residue was extracted from milk using an extraction procedure based on the QuEChERS methodology with some modifications.^16^ In this regard, 2.0 g of milk sample was weighed in a polypropylene tube followed by the addition of 20 mL of acetonitrile (containing 1% acetic acid). Subsequently, the mixture was stirred in a shaker for 30 s. Then, 1.0 g of sodium chloride and 2.0 g of anhydrous sodium sulfate were added and the sample was vortexed for 5 min using a vortex mixer (Dragon Lab instrument, Beijing, China). Following centrifugation of samples at 14 000 × *g* and 10 °C for 5 min, 10 mL of the supernatant was separated and transferred to a micro-tube and treated with 100 mg of sorbent C18 to remove fatty acids. The mixture was oscillated slowly for about 5 min and centrifuged again under the same conditions described above for 5 min. Then, 5 mL of clear supernatant was transferred into a conical bottom glass. Finally, the sample was evaporated on a Turbo-Vap II station (Zymark, Hopkinton, MA, USA) under the nitrogen flow at 50 °C. The remaining residue was dissolved in 1 mL of acetonitrile by mixing in a vortex mixer for 1 min. The same procedure was carried out on 20 mL of water to compare the stability of spiramycin in the milk matrix to that of water. For quantitation by LC-MS/MS, samples were filtered through a 0.22 μm PTFE syringe filter, and 20 μL of the final test solution was injected onto the LC column. The same procedure was conducted on 20 mL of water to compare the stability of spiramycin in the milk matrix to that of water.

#### 2.2.2 Nuclear magnetic resonance spectroscopy

To prove the location of the solvent addition, the stock solution of spiramycin was prepared in DMSO-d_6_ (8 mg mL^−1^) for ^1^H NMR measurement. Then by adding 100 μL of D_2_O to the same solution, the ^1^H NMR spectrum was recorded again. ^1^H NMR spectra were recorded at 25 °C using a 400 MHz Bruker Avance III HD spectrometer (Bruker Daltonics GmbH, Bremen, Germany).

## 3. Results and discussion

### 3.1. Solvent addition into spiramycin

**Fig. 2:**
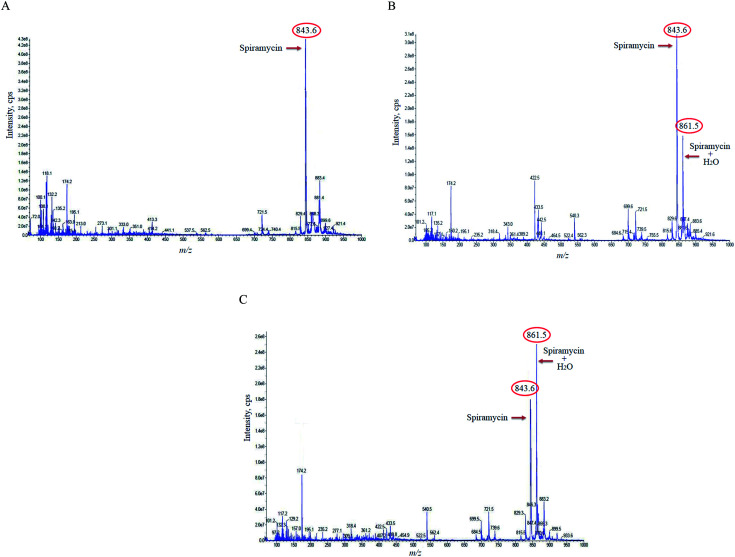
The mass spectra of spiramycin aqueous standard solution 0 min (A), 30 min (B), and 10 h (C) following preparation of the standard solution. Spiramycin: 843.60 *m*/*z*; H_2_O-bound spiramycin: 861.5 *m*/*z*.

In the case of the pure standard aqueous solution, an additional peak in the mass spectrum of spiramycin was observed in the acquired mass spectra which did not correspond to routine adducts such as H-adduct, sodium adduct, and or potassium adduct. At first, the mentioned mass seemed to be an impurity, but due to the high peak intensity of the observed mass in the full-scan mode and considering the mass difference between the molecular weight of spiramycin and the *m*/*z* value of this peak (18 mass units) we hypothesized that the added mass may be due to the addition of an H_2_O molecule to the spiramycin molecule. In this regard, the masses of spiramycin and H_2_O-bound spiramycin were monitored at different time intervals. The obtained mass spectra are illustrated in Fig. 2A–C. As shown in Fig. 2A, in a freshly prepared standard solution no H_2_O molecule is bound to the spiramycin molecule while as time passes the signal of spiramycin (molecular weight: 843.60 g mol^−1^ in the positive mode) decreases, and an increment in the signal of H_2_O-bound spiramycin (molecular weight: 861.5 g mol^−1^) is observed (Fig. 2B and C).

**Fig. 3:**
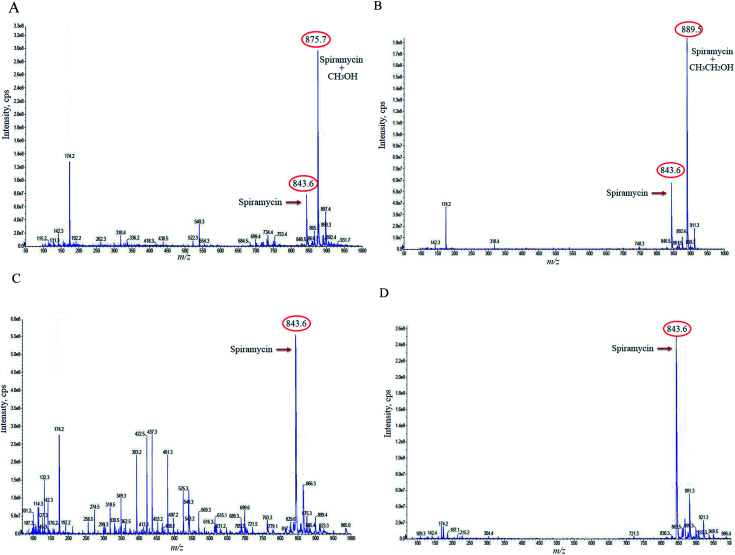
The mass spectra of methanol (A), ethanol (B), acetonitrile (C), and dimethyl sulfoxide (D) reference solutions. Spiramycin: 843.60 *m*/*z*; methanol-bound spiramycin: 875.7 *m*/*z*; ethanol-bound spiramycin: 889.5 *m*/*z*.

Based on the chemical structure of spiramycin, it was hypothesized that the added mass belongs to the water molecule as a protic solvent that is added to the formyl group of spiramycin. To prove this hypothesis, the mass spectra of spiramycin dissolved in protic solvents (methanol, ethanol) and aprotic solvents (acetonitrile, and DMSO) are presented in Fig. 3A, B, 3C, and 3D, respectively. As illustrated in Fig. 3A and B, methanol (molecular weight: 32.04 g mol^−1^) and ethanol (molecular weight: 46.07 g mol^−1^) molecules add to the spiramycin molecule (molecular weight: 843.6 g mol^−1^ in the positive mode) and shift the molecular mass of spiramycin to 875.7 g mol^−1^ and 889.5 g mol^−1^, respectively while there is no evidence for the addition of acetonitrile (molecular weight: 41.05 g mol^−1^) and DMSO (molecular weight: 78.13 g mol^−1^) molecules to the spiramycin structure.

### 3.2. The location of the solvent addition

**Fig. 4:**
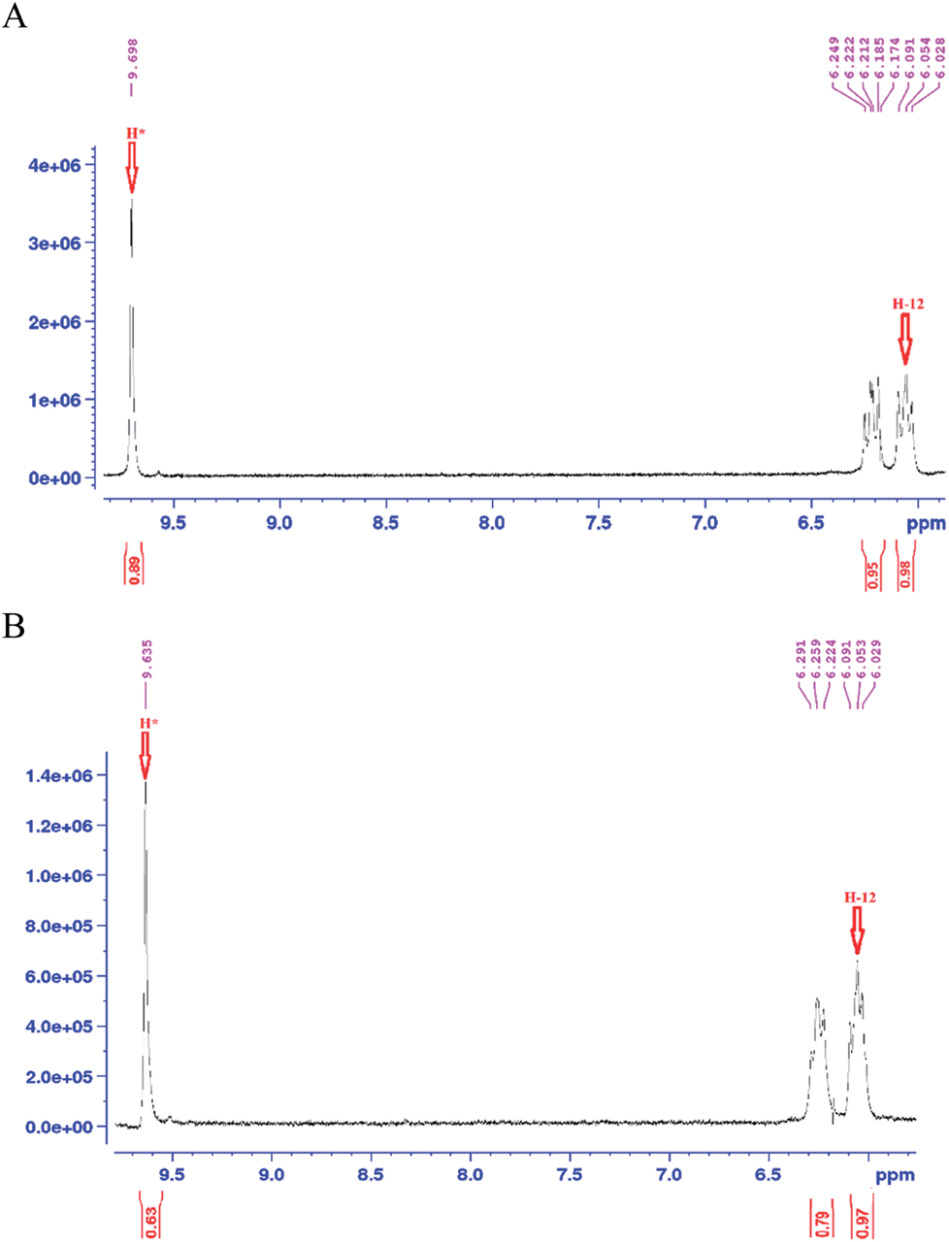
The ^1^H NMR spectrum of spiramycin pure standard dissolved in d_6_-dimethyl sulfoxide (A) and the ^1^H NMR spectrum of the same solution following the addition of 100 μL of deuterium oxide (D_2_O) (B).

As mentioned earlier, a mass equivalent to the molecular mass of spiramycin plus solvent molecules was observed in the mass spectra when the spiramycin was dissolved in protic solvents. Based on the chemical structure of spiramycin, it was postulated that the added mass could be due to the addition of solvent molecules to spiramycin. To prove the location of the solvent addition, the NMR spectroscopy was carried out. The ^1^H NMR spectrum of spiramycin dissolved in d_6_-DMSO and d_6_-DMSO + D_2_O are shown in Fig. 4A and B, respectively. The spiramycin molecule is reactive from the aldehyde moiety and the lactone ring (Fig. 1) and if the solvent is added to the aldehyde moiety, the aldehyde proton integration will decrease in the ^1^H NMR spectrum. It was observed that the integration of aldehyde hydrogen (H* in Fig. 1) compared to the integration of C12-connected hydrogen (H-12) decreases from 90% in d_6_-DMSO to 65% in d_6_-DMSO + D_2_O, which proves the reaction of spiramycin on the aldehyde group with the added D_2_O (or water molecule).

**Fig. 5:**
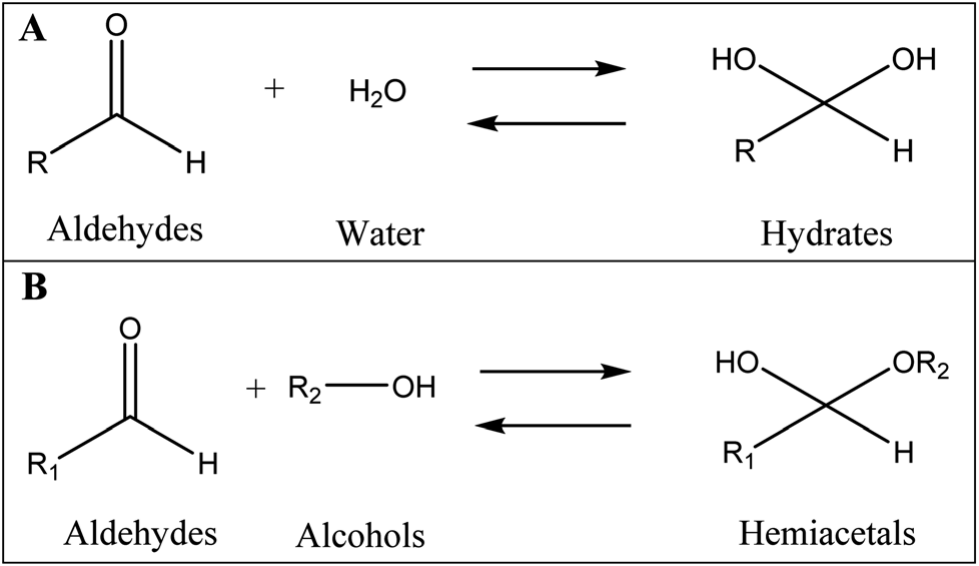
The reaction of aldehydes with water (A) and alcohols (B) to respectively produce hydrates and hemiacetals.

**Fig. 6:**
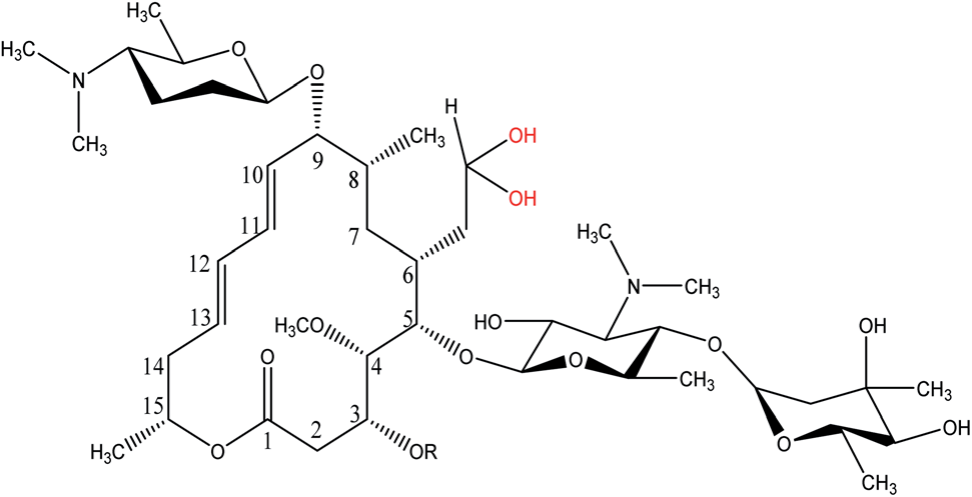
Hydrated spiramycin from the aldehyde region.

It is well documented that aldehydes react with water to give 1,1-geminal diols known as hydrates. Hydrates are not stable enough to be isolated as an independent compound and they exist in equilibrium with the aldehyde form (Fig. 5A). The reaction is a typical nucleophilic attack of the water molecule on the carbonyl group. Similar to this reaction, is the reaction of alcohols with aldehyde which results in hemiacetals. Alcohols just like water are nucleophilic reagents and thus are able to react with aldehydes (Fig. 5B). Aldehydes can also undergo addition reactions with primary and secondary amines and thiols to form a carbinolamine and hemithioacetal respectively. Fig. 6 depicts the hydration of the spiramycin aldehyde group.

The lactone molecule is electrophilic at the carbonyl of the lactone ring. The ring-opening reaction is suggested to proceed through a nucleophilic reaction involving the cleavage of the CO–O bond. A nucleophile in the presence of a Brønsted acid attacks the carbonyl and the CO–O cleavage may lead to the ring-opening of the lactone.^17^

### 3.3. Stability of spiramycin in the milk matrix

**Fig. 7:**
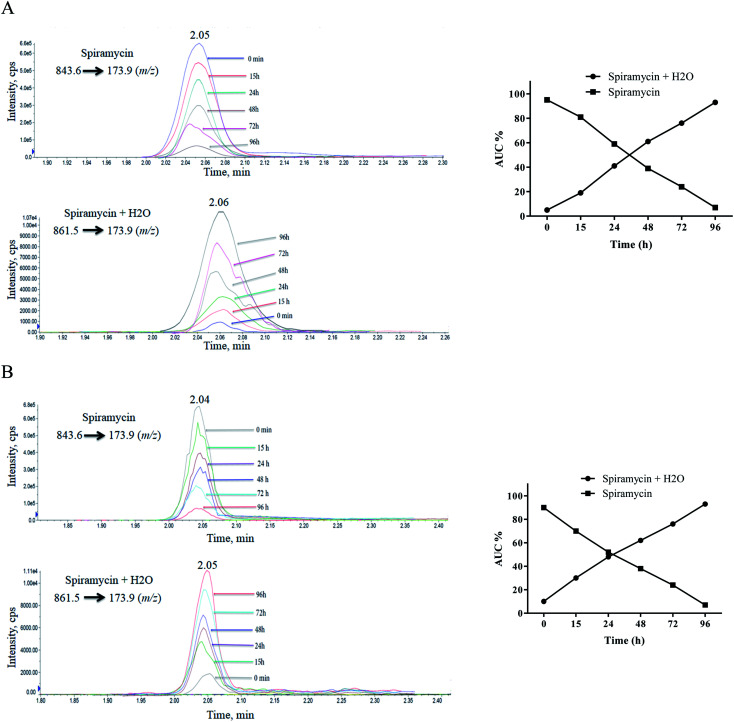
Time lapsed extracted mass spectra of spiramycin and its H_2_O-added form in water (A) and milk sample (B).

Since a maximum residue limit of 200 μg kg^−1^ for spiramycin has been set for milk by Codex Alimentarius,^18^ milk samples are required to be analyzed for spiramycin residue in them. In this regard, the stability of spiramycin was further evaluated in the milk samples at different time intervals following the spiking of the milk sample. The same procedure was conducted on the water to compare the stability of spiramycin in the milk matrix to that of water. The extracted mass spectra of spiramycin spiked in water and milk sample are presented in Fig. 7A and B, respectively. As shown in these figures, as time passes, the area under the curve (AUC) of spiramycin decreases in both the aqueous solution and the spiked milk sample while an increase in the AUC of H_2_O-bound spiramycin is observed in both samples. After 96 h, more than 90% of spiramycin is converted to H_2_O-bound spiramycin either in the spiramycin aqueous solution or in the spiked milk sample.

Spiramycin will undergo a hydration reaction in aqueous matrices and therefore its molecular weight is changed from 843 to 861. The new molecule has an 18 dalton mass difference with spiramycin and therefore it will be invisible for the MRM ion transition which is set for monitoring of spiramycin. To fix this shortcoming, two ion masses including 843.6 *m*/*z* and 861.5 *m*/*z* should be considered as the parent ions that are related to spiramycin and H_2_O-bound spiramycin molecules, respectively.

In most of the previous studies conducted for the determination of spiramycin residue in milk samples, the pure standard solutions of spiramycin were prepared in methanol or water,^15,16,18–35^ and in a few studies, the standard solutions were prepared in acetonitrile.^36–38^ The results of our study revealed that there is a possibility for the intrusion of protic solvents into the spiramycin molecules during the preparation of standard solutions such as stock solutions and working standard solutions or even during the preparation of the sample. For this reason, aprotic solvents such as acetonitrile and DMSO are proposed to be used for the preparation of standard solutions especially when the standard solutions are supposed to be stored for a while. Besides, two ion transitions (843.6 *m*/*z* to 173.9 *m*/*z* and 861.5 *m*/*z* to 173.9 *m*/*z*) should be considered for the accurate quantification of spiramycin residue in food samples such as milk. The first ion transition is used for the quantification of spiramycin molecules and the second one for the quantification of H_2_O-bound spiramycin molecules. This will result in a more precise, accurate, and valid quantification of spiramycin in such kinds of matrices.

## 4. Conclusion

During the preparation of spiramycin standard solutions and even during sample preparation, protic solvents (*e.g.*, water, ethanol, and methanol) will be added into the spiramycin molecules from the aldehyde group location while there is no chance for the addition of aprotic solvents (*e.g.*, acetonitrile and DMSO). However, the kinetic of the conversion reaction is not clear and it is expected to be dependent on time, temperature and spiramycin concentration, *etc.* Based on the obtained results, protic solvents are the most convenient solvents for the preparation of spiramycin standard solutions especially when the prepared solutions are not used instantly. In addition, spiramycin will undergo a hydration reaction in matrices with high content of water such as milk samples. Therefore, the addition of H_2_O molecules to the spiramycin aldehyde group should be considered for accurate quantification. In this regard, two ion masses including 843.6 *m*/*z* and 861.5 *m*/*z* should be considered as the parent ions that are related to spiramycin and H_2_O-bound spiramycin molecules, respectively. Although this will result in a more precise, accurate, and valid quantification of spiramycin in such kinds of matrices, the detector response to spiramycin and spiramycin hydrate may not be equal due to their different proton affinity in the electrospray ionization system. Therefore, our results give a “heads-up” regarding this possible neglected fact which may be a source of error in the quantification of spiramycin. It is also necessary to study the possibility of protic solvents' intrusion into other drugs with aldehyde functional groups such as neospiramycin, tylosin, and carbomycin using LC-MS/MS.

## Conflicts of interest

There are no conflicts to declare.

## Supplementary Material
